# Real-world multicentre cohort of first-line pembrolizumab alone or in combination with platinum-based chemotherapy in non-small cell lung cancer PD-L1 ≥ 50%

**DOI:** 10.1007/s00262-022-03359-2

**Published:** 2023-01-24

**Authors:** E. Pons-Tostivint, P. Hulo, V. Guardiolle, L. Bodot, A. Rabeau, M. Porte, S. Hiret, P. Demontrond, H. Curcio, A. Boudoussier, R. Veillon, M. Mayenga, C. Dumenil, T. Chatellier, P. A. Gourraud, J. Mazieres, J. Bennouna

**Affiliations:** 1 Centre Hospitalier Universitaire Nantes, Medical Oncology, Nantes University, 44000 Nantes, France; 2 Medical Oncology Unit, Clinique Mutualiste de L’Estuaire, Saint-Nazaire, France; 3 Centre Hospitalier Universitaire Nantes, Institute of Health and Medical Research, Santé Publique, Clinique Des Données, Inserm CIC 1413, Centre Hospitalier Universitaire de Nantes, Nantes University, 44000 Nantes, France; 4 Thoracic Oncology Department, Hôpital Larrey, CHU Toulouse, 31000 Toulouse, France; 5 Department of Medical Oncology, Comprehensive Cancer Center, Institut de Cancérologie de L’Ouest, Saint-Herblain, France; 6 Department of Pneumology, Centre François Baclesse, Caen, France; 7 Department of Pneumology, University Hospital of Bordeaux, Pessac, France; 8 Department of Medical Oncology, Hospital Foch, Suresnes, France; 9 Department of Respiratory Diseases and Thoracic Oncology, APHP-Hopital Ambroise Pare, 92100 Boulogne-Billancourt, France

**Keywords:** Non-small cell lung cancer, First-line, Immunotherapy, Chemo-immunotherapy

## Abstract

**Introduction:**

Pembrolizumab alone (IO-mono) or in combination with platinum-based chemotherapy (CT-IO) is first-line standard of care for advanced non-small cell lung cancer (NSCLC) patients with PD-L1 ≥ 50%. This retrospective multicentre study assessed real-world use and efficacy of both strategies.

**Methods:**

Patients with advanced NSCLC PD-L1 ≥ 50% from eight hospitals who had received at least one cycle of IO-mono or CT-IO were included. Overall survival (OS) and real-word progression-free-survival were estimated using Kaplan–Meier methodology. Cox proportional hazards regression models were used to estimate hazard ratios (HRs) and 95% CIs, and a Cox model with inverse propensity treatment weighting was carried out.

**Results:**

Among the 243 patients included, 141 (58%) received IO-mono and 102 (42%) CT-IO. Younger patients, those with symptomatic disease and brain metastases were more likely to be proposed CT-IO. With a median follow-up of 11.5 months (95% CI 10.4–13.3), median OS was not reached, but no difference was observed between groups (*p* = 0.51). Early deaths at 12 weeks were 11% (95% CI 4.6–16.9) and 15.2% (95% CI 9.0–20.9) in CT-IO and IO groups (*p* = 0.32). After adjustment for age, gender, performance status, histology, brain metastases, liver metastases and tobacco status, no statistically significant difference was found for OS between groups, neither in the multivariate adjusted model [HR 1.07 (95% CI 0.61–1.86), *p* = 0.8] nor in propensity adjusted analysis [HR 0.99 (95% CI 0.60–1.65), *p* = 0.99]. Male gender (HR 2.01, *p* = 0.01) and PS ≥ 2 (HR 3.28, *p* < 0.001) were found to be negative independent predictive factors for OS.

**Conclusion:**

Younger patients, those with symptomatic disease and brain metastases were more likely to be proposed CT-IO. However, sparing the chemotherapy in first-line does not appear to impact survival outcomes, even regarding early deaths.

**Supplementary Information:**

The online version contains supplementary material available at 10.1007/s00262-022-03359-2.

## Introduction

Lung cancer is the leading cause of cancer-related death in Europe and worldwide. Over the past decade, non-small cell lung cancer (NSCLC) without oncogenic addiction has undergone a major therapeutic paradigm shift with the development of immune checkpoint inhibitors (ICIs). This new therapeutic class, most often used in combination with platinum-based chemotherapy improves overall survival (OS), progression-free-survival (PFS) and objective response rate (ORR) in non-pre-treated NSCLC. In the subset of advanced NSCLC patients harbouring high Programmed Death-Ligand 1 (PD-L1) with a tumour proportion score (TPS) ≥ 50%, pembrolizumab alone is an established first-line option based on the KEYNOTE-024 [1]. These results were confirmed in this pre-defined population in the KEYNOTE-042 trial with the same anti-PD1 antibody but also with atezolizumab and cemiplimab [2–4]. The updated results of the KEYNOTE-024 reported an unprecedented 5-year OS of 31.9% with pembrolizumab *versus* 16% with platinum-based chemotherapy [5]. Despite major improvements in survival, predicting who will respond or not to ICIs has proved to be difficult. So far, PD-L1 is the only biomarker recommended by the ESMO and NCCN guidelines [6, 7]. The threshold value of 50% for PD-L1 expression on tumour cells is the only predictive biomarker available in clinical practice for pembrolizumab as single agent in non-pre-treated advanced NSCLC [8]. However, a proportion of patients have no benefit from this chemotherapy-free strategy. Moreover, ICI alone can lead to hyperprogressive disease defined as rapid tumour growth after the beginning of treatment. This deleterious effect has been reported in almost 14% of advanced NSCLC treated with ICIs, associated with a lower OS [9, 10]. Consequently, the use of platinum-based chemotherapy combined with ICI is the strategy currently available to avoid this pattern of response and/or early failure of ICI alone, even if both strategies seem to provide similar long-term OS [11, 12]. The toxicity profile of each strategy should also be incorporated in the therapeutic decision, essentially for elderly and/or frail patients.

Therefore, one of the daily-practice clinical questions remains to propose the optimal first-line treatment for patients with advanced NSCLC PD-L1 ≥ 50%. So far, no trial has provided a direct comparison between single-agent anti-PD1 or PD-L1 antibodies *versus* platinum-based chemotherapy plus ICI. Consequently, both options are available in accordance with the guidelines [6, 7]. The prognostic and predictive baseline factors related to the patient (such as age, gender, performance status (PS), smoking history) and tumours (such as metastatic sites or co-mutations) have been evaluated [13–17]. Several retrospective analyses showed that not smoking, PS 2, bone and liver metastases are independent predictive negative factors for OS in advanced NSCLC patients with PD-L1 ≥ 50% treated with first-line pembrolizumab [15, 16, 18].

Based on these data, there is a need to better select patients with NSCLC expressing PD-L1 ≥ 50% eligible for ICI monotherapy. Here, we report the results of a French retrospective multicentre study evaluating patients and disease characteristics associated with physician’s choices of single-agent pembrolizumab or in combination with platinum-based therapy. We compared the real-world effectiveness of these therapeutic strategies with a focus on early-death events.

## Patients and methods

### Study design and data source

This is a French retrospective multicentre study including all consecutive eligible patients with untreated advanced NSCLC. Patients were eligible if they received at least one cycle with pembrolizumab alone (IO group) or combined with a platinum-doublet chemotherapy (CT-IO group). The chemotherapy backbone was carboplatin/cisplatin with pemetrexed for non-squamous or carboplatin with paclitaxel for squamous histology. This study was conducted from December 2019 for non-squamous NSCLC and June 2020 for squamous NSCLC, which corresponds to the reimbursement date in France for each subtype, respectively. Patients were included until July 2021. Overall, eight French hospitals were involved, including University Hospitals, Cancer Centres and Public and Private Hospitals. Other eligibility criteria were as follows: histological or cytological confirmed stage III (not amenable to curative treatment) or stage IV NSCLC, no participation in clinical trials, neither EGFR mutations nor ALK or ROS-1 translocations. All clinical, radiological, biological variables, and treatment information, were manually extracted from medical records. Corticosteroid use was defined as any systemic corticosteroid therapy (excluding premedication) within 7 days before or at first-systemic treatment administration. The PD-L1 expression was analysed by immunohistochemistry on tumour cells as recommended by guidelines [6]. This study was conducted in accordance with the local regulations and was approved by the local independent ethics committee.

### Study endpoints

The primary endpoint was OS, defined as the time from the start of the first-line treatment of interest to the date of death due to any cause, or was censored on the day of cut-off. Early death was defined as death within 12 weeks from the start of treatment. Secondary endpoints were real-world PFS (rwPFS), defined as the time from the start of treatment to death or disease progression reported in radiology reports or clinical assessment. Patients with missing information for the date of last clinical note, and no progression, were excluded from the rwPFS analysis. Each patient underwent a baseline full body computed tomography scan (CT); baseline brain imaging (CT or magnetic resonance) was performed according to the participating centres’ local clinical practice and to the respective national guidelines. Real-world disease control rate (rwDCR) was defined by patients displaying stable disease (SD), partial response (PR) or complete response (CR). The patients were assessed with radiological imaging in clinical practice, according to the local monitoring requirements, in general every three months. The index date was defined as the date of first-line treatment initiation, and patients were followed up until the last date of follow-up or end of study date (November 2021), whichever occurred first.

### Statistical analysis

Descriptive analyses were used to characterize the most relevant clinical variables. Categorical parameters were explored using the Chi-square test or Fisher’s exact test. Continuous variables were compared using Student's t test or the Mann–Whitney *U* test depending on whether they showed a normal distribution. OS and rwPFS were calculated using the Kaplan–Meier method and log-rank test. Cox proportional hazards regression models were used to estimate hazard ratios (HRs) and 95% confidence intervals (CIs), and a Cox model with inverse propensity treatment weighting was carried out to reduce potential bias resulting from confounding factors’ inequalities. The study cut-off date was November 2021. Median period of follow-up was computed according to the reverse Kaplan–Meier method. All analyses were conducted on the R software (version 3.3.0).

## Results

### Patients and disease characteristics

A total of 243 patients were included. Among them, 141 (58.0%) received IO, and 102 (42.0%) a combination of CT-IO. Patient and disease characteristics are listed in Table 1. Most of the patients were men (58.2% in IO, 55.9% in CT-IO, *p* = 0.793), with a PS of 0–1 (75.2% in IO, 82.3% in CT-IO, *p* = 0.209), and non-squamous histology (81.6% in IO, 95.1% in CT-IO, *p* = 0.002). The PD-L1 level was equally distributed between both groups (PD-L1 ≥ 90% in 41.8% in IO and 48.0% in CT-IO, *p* = 0.362). Patients who received IO alone were more likely to be aged 65 years or more (63.1% in IO, 28.4% in CT-IO, *p* < 0.001). The frequency of asymptomatic disease was higher in the IO-group (27.7% vs. 11.8% in CT-IO, *p* = 0.002). More patients who received IO had a locally advanced stage (18.4% vs. 5.9% in CT-IO, *p* = 0.004) or only one metastatic site (43.5% vs. 34.3% in CT-IO, *p* = 0.006). No difference was reported for corticosteroid use at diagnosis (13.8% had ≥ 10 mg/day in IO, 22.2% in CT-IO, *p* = 0.215). Brain metastases were present at diagnosis in 23.4% and 39.4% of patients in the IO and CT-IO groups, respectively (*p* = 0.003). There was no difference by groups regarding KRAS, TP53 and BRAF status for non-squamous histology (data not shown). No differences were found for baseline LDH, albumin, haemoglobin; however, data were missing for a subset of patients (supplementary Table 1).

**Table 1:** Baseline patient and tumour characteristics by treatment group

Characteristics, *n* (%)	IO (*n* = 141)	CT-IO (*n* = 102)	*p* value
*Patient characteristics*			
Gender			
Men	82 (58.2)	57 (55.9)	
Women	59 (41.8)	45 (44.1)	0.793
Age group, years			
< 65	52 (36.9)	73 (71.6)	
≥ 65y	89 (63.1)	29 (28.4)	< 0.001
Age, years			
Median [range]	68 [47–92]	61 [35–81]	< 0.001
ECOG performance status			
0–1	106 (75.2)	84 (82.3)	
≥ 2	35 (24.8)	18 (17.6)	0.209
Smoking status			
Never	12 (8.8)	3 (3.0)	
Current or past	124 (91.2)	96 (97.0)	
Missing	5	3	0.104
Medications at diagnosis			
None	16 (11.8)	20 (19.6)	
1–3	53 (39.0)	41 (40.2)	
> 3	67 (49.3)	41 (40.2)	
Missing	5	0	0.185
Weight loss at diagnosis			
Yes	36 (32.7)	36 (39.1)	
No	74 (67.3)	56 (60.9)	
Missing	31	10	0.378
Symptomatic disease at diagnosis			
Yes	102 (72.3)	90 (88.2)	
No	39 (27.7)	12 (11.8)	0.002
Corticoids at diagnosis			
≥ 10 mg/day	19 (13.8)	22 (22.2)	
< 10 mg/day	2 (1.4)	1 (1.0)	
None	117 (84.8)	76 (76.8)	
Missing	3	3	0.215
Site of first treatment administration			
Ambulatory	119 (84.4)	73 (71.6)	
Hospitalization	22 (15.6)	29 (28.4)	0.017
*Baseline tumour characteristics*			
Histology			
Non-squamous	115 (81.6)	97 (95.1)	
Squamous	26 (18.4)	5 (4.9)	0.002
PD-L1 status			
90–100	59 (41.8)	49 (48.0)	
50—89	82 (58.2)	53 (52.0)	0.362
Tumour stage			
Locally advanced	26 (18.4)	6 (5.9)	
Metastatic	115 (81.6)	96 (94.1)	0.004
Number of metastatic site	*N* = 115	*N* = 96	
1	50 (43.5)	33 (34.3)	
≥ 2	65 (56.5)	63 (65.6)	0.006
Brain metastases			
Yes	26 (23.4)	37 (39.4)	
No	85 (76.6)	57 (60.6)	
Missing	4	2	0.003
Liver metastases			
Yes	22 (19.1)	16 (16.7)	
No	93 (80.9)	80 (83.3)	1.00
Adrenal metastases			
Yes	28 (24.3)	33 (34.4)	
No	87 (75.6)	63 (65.6)	0.035
Bone metastases			
Yes	41 (35.6)	35 (36.5)	
No	74 (64.3)	61 (63.5)	0.402
Lung metastases			
Yes	34 (29.6)	24 (25.0)	
No	81 (70.4)	72 (75.0)	1.00
Pleural effusion			
Yes	23 (20.0)	21 (21.9)	
No	92 (80.0)	75 (78.1)	0.506
*N* patients			
First treatment of brain metastases	26	37	
Yes	17 (65.4)	21 (56.8)	
No	9 (34.6)	16 (43.2)	0.475

*IO* immunotherapy, *CT-IO* chemotherapy plus immunotherapy, *ECOG* Eastern Cooperative Oncology Group

### Overall survival

**Fig. 1:**
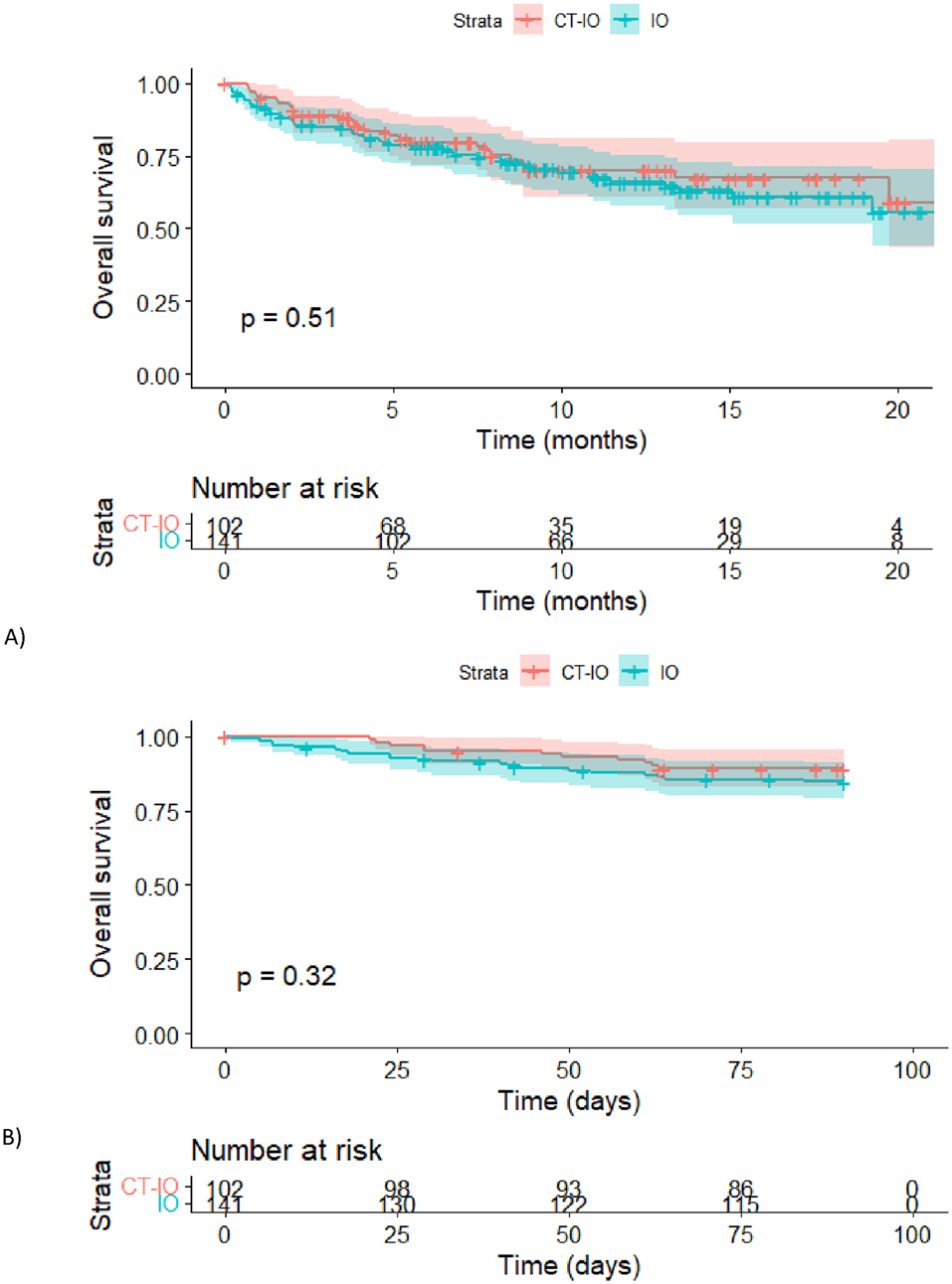
Overall survival with 95% CIs. **A** Kaplan–Meier plot for OS by treatment group. **B** Kaplan–Meier plot for OS with a focus on early-death events within the first 12 weeks, by treatment group

The median follow-up was of 11.5 months in the whole cohort (95% CI 10.4–13.3), 12.6 months [95% CI 11.1–13.9] for IO and 9.97 months [95% CI 9.0–13.1] for CT-IO. At the data cut-off, 46 deaths and 27 deaths were observed in the IO and CT-IO groups, respectively. Seventeen (12.0%) and 11 (10.8%) patients stopped the treatment for toxicity in the IO and CT-IO groups, respectively. Median OS was not reached (NR) (*p* = 0.51) (Fig. 1A). Estimated 12-month OS rate was 66.1% [95% CI 58.0–75.3] for IO and 70.2% [95% CI 60.7–81.1] for CT-IO. Early deaths within 12 weeks after the first injection were observed for 10.8% (11/102) and 14.9% (21/141) of patients in the CT-IO and IO groups, respectively, with no difference between groups (p = 0.32) (Fig. 1B).

In the multivariate adjusted model on age, gender, PS, histology, brain metastases, liver metastases and tobacco status, no statistically difference was found between IO and CT-IO neither for OS (HR 1.06, 95% CI 0.61–1.83, *p* = 0.84) nor for early-death events (HR 1.40, 95% CI 0.58–3.33, *p* = 0.45) (Table 2). After propensity adjustment, the same results were found for OS (HR 1.00, 95% CI 0.60–1.6, *p* = 0.99) and for early-death events (HR 0.91, 95% CI, 0.40–2.07, *p* = 0.82). Male gender (HR 2.01, CI 95% 1.16–3.50, *p* = 0.01) and PS ≥ 2 (HR 3.28, 95% CI 1.95–5.51, *p* < 0.001) were found to be negative independent predictive factors for OS.

**Table 2:** Multivariate analyses of prognostic factors associated with OS and early deaths within 12 weeks

	Overall survival		Early deaths within 12 weeks	
	Multivariate analyses Adjusted HR [95% CI]	*p* value	Multivariate analyses Adjusted HR [95% CI]	*p* value
Age, years				
< 65	1.00		1.00	
≥ 65	1.17 [0.69–2.01]	0.55	1.01 [0.45–2.27]	0.98
Gender				
Women	1.00		1.00	
Men	2.01 [1.16–3.50]	0.01	3.77 [ 1.47–9.68]	0.006
ECOG PS				
0–1	1.00		1.00	
≥ 2	3.28 [1.95–5.51]	< 0.001	3.98 [1.86–8.50]	< 0.001
Histology				
Squamous	1.00		1.00	
Non-squamous	1.74 [0.73–4.15]	0.21	0.61 [0.22–1.71]	0.34
Brain Metastases				
No	1.00		1.00	
Yes	1.21 [0.69–2.11]	0.51	2.18 [0.97–4.92]	0.06
Liver Metastases				
No	1.00		1.00	
Yes	1.68 [0.94–3.0I3]	0.08	0.90 [0.34–2.34]	0.82
Smoking status				
Current/former	1.00		1.00	
Never	0.69 [0.22–2.13]	0.51	1.10 [0.13–9.12]	0.93
Treatment				
CT-IO	1.00		1.00	
IO	1.06 [0.61–1.83]	0.84	1.40 [0.58–3.33]	0.45

*IO* immunotherapy, *CT-IO* chemotherapy plus immunotherapy, *ECOG PS* Eastern Cooperative Oncology Group Performance Status, *HR* Hazard ratio

### Real-world progression free survival, response rate, and subsequent therapies

**Fig. 2:**
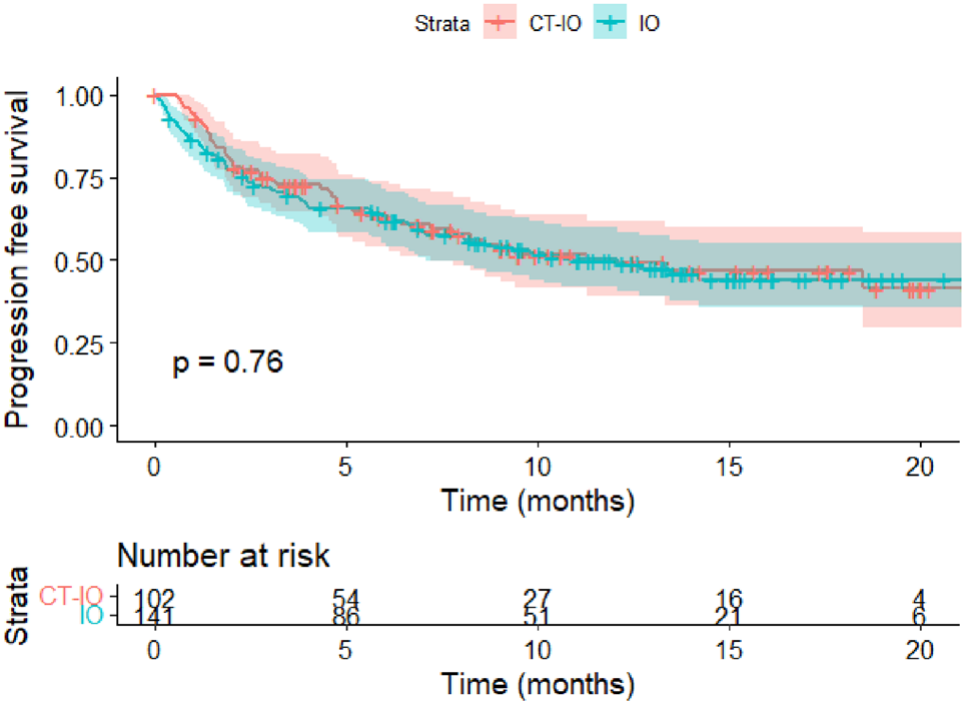
Real-word progression-free-survival by treatment group with 95% CIs

At the data cut-off, 46/102 (45.1%) patients had progressed with CT-IO and 69/141 (48.9%) patients with IO. Median rwPFS was 11.3 (95% CI 7.2–NR) for CT-IO and 10.6 months (95% CI 7.1–NR) for IO, respectively (*p* = 0.76) (Fig. 2). Estimated 12-month PFS rate was 50.0% (95% CI 41.9–59.7) for IO and 49.3% (95% CI 39.2–61.9) for CT-IO. After adjustment on age, gender, PS, histology, brain metastases, liver metastases and tobacco status, no statistically difference for rwPFS was found between IO and CT-IO, neither in the multivariate adjusted model (HR 0.94, 95% CI 0.61–1.45, *p* = 0.78) (Table 3) nor in the propensity adjusted analysis (HR 1.11, 95% CI 0.75–1.66, *p* = 0.59).

**Table 3:** Multivariate analyses of prognostic factors associated with real-world PFS

	Progression free-survival	
	Multivariate analyses Adjusted HR [95% CI]	*p* value
Age, years		
< 65	1.00	
≥ 65	1.11 [0.72–1.71]	0.64
Gender		
Women	1.00	
Men	2.26 [1.44–3.55]	< 0.001
ECOG PS		
0–1	1.00	
≥ 2	2.31 [1.48–3.61]	< 0.001
Histology		
Squamous	1.00	
Non-squamous	1.78 [0.87–3.62]	0.11
Brain Metastases		
No	1.00	
Yes	1.02 [0.63–1.62]	0.94
Liver Metastases		
No	1.00	
Yes	1.52 [0.92–2.52]	0.10
Smoking status		
Current/former	1.00	
Never smoker	0.38 [0.17–0.84]	0.02
Treatment		
CT-IO	1.00	
IO mono	0.94 [0.61–1.45]	0.78

*IO* immunotherapy, *CT-IO* chemotherapy plus immunotherapy, *ECOG PS* Eastern Cooperative Oncology Group Performance Status, *HR* Hazard ratio

Real-world response rate was assessable for 132/141 patients in the IO group, and 97/102 patients in the CT-IO group (Supplementary Table 2). The best objective response rate was 59.8% with combination therapy, and 47.1% with IO. Real-world DCR was similar in both groups (78.3% in CT-IO and 68.9% in IO mono, *p* = 0.133). There were no clear differences regarding sites of relapse between groups. Among patients with progressive disease, proportion, and duration of second-line treatment was similar between groups (Supplementary Table 3).

### Subgroup analyses

**Fig. 3:**
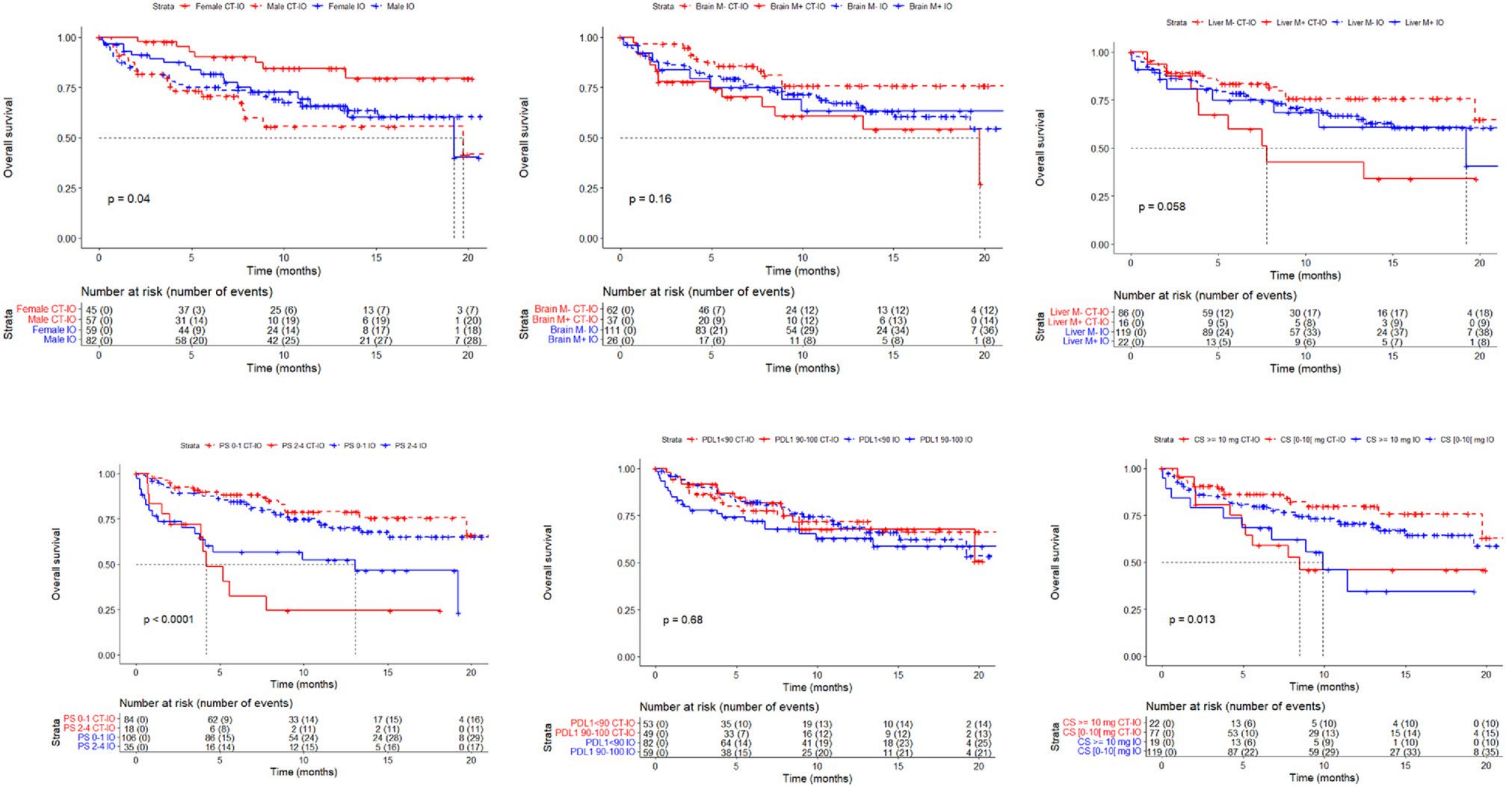
Kaplan–Meier plot for overall survival, by treatment group and **A** gender, **B** brain metastasis (at baseline), **C** liver metastasis (at baseline), **D** PS status (at baseline), **E** PD-L1 status, **F** Corticosteroids (at baseline)

**Fig. 4:**
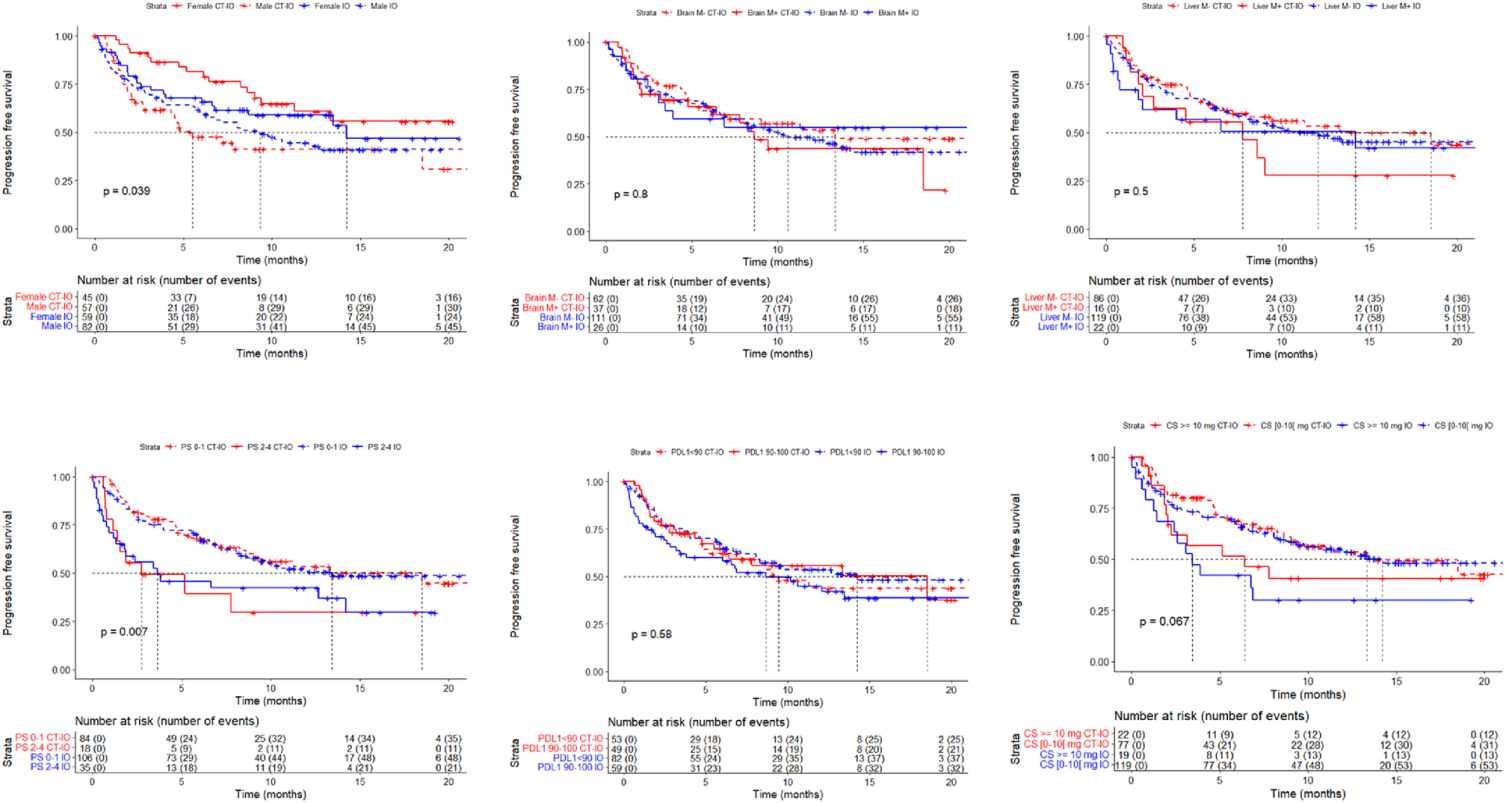
Kaplan–Meier plot for rw-progression-free survival, by treatment group and **A** gender, **B** brain metastasis (at baseline), **C** liver metastasis (at baseline), **D** PS status (at baseline), **E** PD-L1 status, **F** corticosteroids (at baseline)

We performed unplanned subgroup analyses for OS and rwPFS according to gender, metastatic sites (brain and liver), PS, level of PD-L1 expression and corticosteroids at first cycle (Figs. 3 and 4). No significant treatment effect differentiation by brain metastases, liver metastases, and PD-L1 status on OS and rwPFS was found. Corticosteroids over 10 mg/day and PS ≥ 2 were associated with a worse OS and rwPFS, whatever the first-line treatment. Gender was also associated with survival outcome difference. Women treated with IO and CT-IO had a median PFS of 14.2 months [95% CI 6.8–NR] and NR [95% CI 11.2–NR], and men had, respectively, a median PFS of 9.3 [95% CI 5.9–NR] and 5.5 [95% CI 4.3–NR]. HR for OS between IO and CT-IO was 0.77 [95% CI 0.43–1.36] for men, and 2.45 [95% CI 1.02–5.89] for women. These analyses were only exploratory due to the design of our study.

## Discussion

In this large multicentre real-life cohort involving NSCLC patients with PD-L1 ≥ 50%, we observed that physicians were more likely to choose a first-line chemo-immunotherapy for younger patients, with symptomatic disease at diagnosis, multi-metastatic involvement, and/or with brain metastasis. Our study showed that after adjustment for known baseline confounder factors, patients treated with first-line single-agent IO had a similar risk of death to those treated with CT-IO, even for early deaths within the first 12 weeks.

The best first-line strategy in advanced NSCLC patients with PD-L1 ≥ 50% is a highly controversial topic. We thought that there is a strong rational to use real-world data to help answer this question. First, to determine factors influencing the physician’s decision in clinical practice, and second, to investigate the efficacy of treatments in subgroups of patients not eligible or under-represented in clinical trials. The real-world data captured in this study showed that up to 24.8% and 17.6% of patients treated with IO and CT-IO had an altered PS at diagnosis ≥ 2, concordant with previous retrospective studies [19–21]. As expected, we found that PS 2 was a pejorative prognostic factor for rwPFS and OS regardless treatment received. It has been demonstrated that patients with a PS of 2 are not good candidates for single-agent ICI in second line or further, but few data are available in a first-line setting [22]. Only one prospective phase 2 trial evaluated PS 2 patients (*n* = 60), demonstrating that pembrolizumab was safe with a durable clinical benefit of 53% in PD-L1 ≥ 50% (*n* = 15) [23]. However, it could be important to consider the determinant of poor PS, as it has been showed that patients initiating pembrolizumab with an PS 2 because of comorbidities had a significantly better prognosis compared with patients whose poor PS was determined by the tumour symptoms [18]. We were not able to differentiate these patients in our study.

Patients with brain metastases represents another group of high importance in clinical practice. Many of the landmark trials that demonstrated the benefit of ICIs in advanced NSCLC excluded brain metastases patients or had limited patients with pre-treated and stable brain metastases [1–4]. Recent pooled analyses have demonstrated a benefit of chemo-immunotherapy and immunotherapy alone, over chemotherapy, with or without baseline brain metastases [24, 25]. In our cohort, nearly 30% of patients had brain metastases at diagnosis, mostly treated with CT-IO (39.4% vs. 23.4% with IO). Presence of brain metastases was not associated with OS in multivariate analysis. However, it was associated with early-death events at the edge of significance (HR 2.18, *p* = 0.06). Additionally, patients with an altered PS and/or brain metastases at baseline received more frequently corticosteroids at baseline, which is correlated with a worse prognosis irrespective of the treatment type.

Female gender was demonstrated to be a prognostic factor in lung cancer largely before the advent of ICIs, mostly related to key prognostic factors such as histology, stage, smoking status, lifestyle characteristics and treatment modalities [26, 27]. Recently, a large Australian cohort confirmed that women survived significantly longer after a diagnosis of lung cancer [28]. As in previous reports, men were older at diagnosis, less likely to be never-smokers, and had more comorbidities. Several authors have suggested that lung cancer in women may have a different natural history not only related to patient factors, but also to immunological differences [26, 29]. Women with advanced lung cancer derived a statistically significant greater benefit from the addition of chemotherapy to ICIs compared to men [30]. A meta-analysis conducted in a first-line setting of NSCLC with a high PD-L1 showed a pooled OS HR comparing single-agent ICI *versus* chemotherapy of 0.59 (95% CI 0.50–0.69) for men and only 0.84 (95% CI 0.64–1.10) for women [17]. In our study, we found men had a worse OS than women in multivariate analyses (HR 2.01, *p* = 0.01). Moreover, women treated with CT-IO seemed to have a longer OS than with IO, whereas men treated with IO had a better OS than those treated with CT-IO. Importantly, we must be careful with these exploratory unadjusted analyses.

Few other studies compared real-world survival outcomes with first-line ICI-based therapy and combination of chemo-immunotherapy in NSCLC patients. To our knowledge, none have been conducted in Europe. Waterhouse et al*.* used the US large electronic health record database named the Flatiron Health oncology database, covering 2016 (the year of first FDA approval for immunotherapy) through June 2020 [21]. They did not provide statistical comparison between IO (*n* = 3041) and CT-IO (*n* = 4271) groups, and there was no selection on PD-L1 status. As in our study, they found that poor PS ≥ 2 was strongly associated with shorter median OS, in both treatment groups. As expected, PD-L1 expression over 50% was strongly associated with OS. A recent comparative study published by Pérol et al*.* evaluated first-line systemic therapy by using the same Flatiron Health oncology database, with a focus on 520 NSCLC patients with high PDL1 expression [31]. However, unlike our study, patients with an altered PS > 1 and squamous histology were excluded. They found that patients receiving IO were older (69% were over 65 years vs. 54% in CT-IO). Brain metastases were reported at diagnosis for 26% and 30% of patients in IO and CT-IO, respectively. As in our study, they observed that sparing chemotherapy in first-line setting did not impact median OS (HR 1.03, 95% CI 0.77–1.39, *p* = 0.83) and rwPFS (HR 1.04, 95% CI 0.78–1.37, *p* = 0.81). Interestingly, a significant differentiation of treatment effect on OS was detected by smoking status, with a significant benefit of CT-IO in never-smoker patients (HR 0.25, 95% CI 0.07–0.83). In our study, the number of non-smoker patients was too low to make any conclusions for this sub-group. The fact that never-smokers, independently of oncogenic alteration, had less benefit from IO monotherapy had been previously suspected [16, 32].

The present study had several limitations. Although observational studies provide rich insights into real-world data, they can only compare outcomes between non-randomized groups of patients with different prognostic factors. There was an imbalance in some clinical and disease characteristics between groups, hence propensity score matched analyses were carried out to limit the potential treatment selection bias. Moreover, retrospective database studies can be marred by errors in recording data and failure to collect some information. For example, biological data were missing for nearly half of the patients, explaining why LIPI (Lung Immune Prognostic Index) score was not mentioned in our analysis [33].

Taking into consideration those weaknesses as described, our study gives an overview of the therapeutic strategies for patients with advanced NSCLC and PD-L1 ≥ 50% proposed in diverse types of French medical institutions (University hospitals, Cancer Centres, Public and Private hospitals). It illustrates that the medical community uses CT-IO or IO-monotherapy without well-identified clear decision-making factors between each group. For example, PD-L1 status, baseline corticosteroid, PS or liver metastases did not show association with a specific treatment strategy. In contrast, we showed that physicians were more prone to propose a first-line combination of chemo-immunotherapy in younger patients, with symptomatic disease at diagnosis, multi-metastatic sites and especially brain metastases. A very large cohort such as the Flatiron Health database offered a high number of patients, but with more missing data that manually collected retrospective cohorts, such as PS (missing for 25% of patients receiving IO in monotherapy in Waterhouse et al.), or PD-L1 status (up to 15%) [21].

In conclusion, we found in our retrospective analysis that sparing chemotherapy in first-line treatment does not appear to impact survival outcomes, even regarding early deaths. The ongoing phase III PERSEE trial in France (NCT04547504) evaluating pembrolizumab versus pembrolizumab plus chemotherapy in PD-L1 ≥ 50% NSCLCs (both histologies), will help to prospectively answer this major question.

## Highlights

The best first-line strategy in advanced NSCLC patients with PD-L1 ≥ 50% is a highly controversial topic.

Physicians proposed CT-IO mostly for younger patients, with symptomatic disease and/or multi-metastatic involvement.

Patients treated with first-line IO had a similar risk of death to those treated with CT-IO, even regarding early deaths.

Male gender and performance status ≥ 2 were found to be negative independent predictive factors for overall survival.

## Supplementary Information

Below is the link to the electronic supplementary material.

Supplementary file1 (DOCX 14 kb)

Supplementary file2 (DOCX 13 kb)

Supplementary file3 (DOCX 13 kb)

## Data Availability

The datasets generated during and/or analysed during the current study are available from the corresponding author on reasonable request.
