# Supplementary material for: Real-world multicentre cohort of first-line pembrolizumab alone or in combination with platinum-based chemotherapy in non-small cell lung cancer PD-L1 ≥ 50%
Source: Cancer Immunol Immunother. 2023 Jan 24;72(6):1881–90. doi: 10.1007/s00262-022-03359-2 (PMC10198917; doi:10.1007/s00262-022-03359-2)
Supplement: Supplementary file 1 — Supplementary file1 (DOCX 14 kb) [file 262_2022_3359_MOESM1_ESM.docx]

Supplementary Table 1: Baseline biological parameters by treatment group.

|  | IO | CT-IO | p-value |
| --- | --- | --- | --- |
| N patients (%) | 141 (58.0) | 102 (42.0) |  |
| LDH   - N - >N - Missing | 29 (59.2)  20 (40.8)  92 | 14 (50.0)  14 (50.0)  74 | 0.481 |
| Albumine (g/L)   - ≥ 30 - < 30 - Missing | 71 (71.0)  29 (29.0)  41 | 61 (80.3)  15 (19.7)  26 | 0.218 |
| Hb (g/dL)   - ≥ 12 - < 12 - Missing | 84 (64.1)  47 (35.9)  10 | 62 (64.6)  34 (35.4)  6 | 1.00 |

LDH, lactate deshydrogenase ; Hb, Haemoglobin ; IO, immunotherapy; CT-IO, chemotherapy plus immunotherapy
