# Supplementary material for: Real-world multicentre cohort of first-line pembrolizumab alone or in combination with platinum-based chemotherapy in non-small cell lung cancer PD-L1 ≥ 50%
Source: Cancer Immunol Immunother. 2023 Jan 24;72(6):1881–90. doi: 10.1007/s00262-022-03359-2 (PMC10198917; doi:10.1007/s00262-022-03359-2)
Supplement: Supplementary file 2 — Supplementary file2 (DOCX 13 kb) [file 262_2022_3359_MOESM2_ESM.docx]

Supplementary Table 2: Response rate by treatment group.

|  | IO | CT-IO | p-value |
| --- | --- | --- | --- |
| Best ORR   - CR - PR - SD - PD - DR - NA | 5 (3.8)  57 (43.3)  29 (22.0)  38 (28.8)  3 (2.3)  9 | 0 (0.0)  58 (59.8)  18 (18.6)  16 (16.5)  5 (5.1)  5 | 0.015 |
| DCR [95% CI] | 68.9 [59.4 – 78.4] | 78.3 [69.1 – 87.6] | 0.133 |

ORR, Objective response rate; CR, complete response ; PR, partial response ; SD, stable disease ; PD, progressive disease ; DR, dissociated response ; NA, not available ; DCR, disease control rate ; IO, immunotherapy; CT-IO, chemotherapy plus immunotherapy
