# Supplementary material for: Real-world multicentre cohort of first-line pembrolizumab alone or in combination with platinum-based chemotherapy in non-small cell lung cancer PD-L1 ≥ 50%
Source: Cancer Immunol Immunother. 2023 Jan 24;72(6):1881–90. doi: 10.1007/s00262-022-03359-2 (PMC10198917; doi:10.1007/s00262-022-03359-2)
Supplement: Supplementary file 3 — Supplementary file3 (DOCX 13 kb) [file 262_2022_3359_MOESM3_ESM.docx]

Supplementary Table 3: Progression site and subsequent therapy by treatment group.

| N patients (%) | IO | CT-IO | p-value |
| --- | --- | --- | --- |
| Among progressors | N=34 | N=54 |  |
| Progression site   - Brain alone - Extra-cerebral - Both | 2 (5.9)  25 (73.5)  7 (20.6) | 3 (5.6)  42 (77.8)  9 (16.7) | 0.792 |
| 2^nd^ line of treatment   - Yes - No | 31 (57.4%)  23 (42.6%) | 23 (67.6%)  11 (32.3%) | 0.160 |
| Median duration of second-line,  months [95% CI] | 3.0 [1.1 – 4.2] | 2.1 [0.8 – 3.3] | 0.096 |
